# Expression of a Small Ubiquitin-Like Modifier Protease Increases Drought Tolerance in Wheat (*Triticum aestivum* L.)

**DOI:** 10.3389/fpls.2019.00266

**Published:** 2019-03-08

**Authors:** Marlon L. le Roux, Karl J. Kunert, Christell van der Vyver, Christopher A. Cullis, Anna-Maria Botha

**Affiliations:** ^1^Department of Genetics, Stellenbosch University, Stellenbosch, South Africa; ^2^Department of Plant and Soil Sciences, Forestry and Agricultural Biotechnology Institute, University of Pretoria, Pretoria, South Africa; ^3^Department of Biology, Case Western Reserve University, Cleveland, OH, United States

**Keywords:** SUMO protease, transgenic wheat, water stress tolerance, drought, RuBisCo, chlorophyll fluorescence, OVERLY TOLERANT TO SALT 1

## Abstract

Post-translation modification of proteins plays a critical role in cellular signaling processes. In recent years, the SUMO (Small Ubiquitin-Like Modifier) class of molecules has emerged as an influential mechanism for target protein management. SUMO proteases play a vital role in regulating pathway flux and are therefore ideal targets for manipulating stress-responses. In the present study, the expression of an *Arabidopsis thaliana* cysteine protease (OVERLY TOLERANT TO SALT-1, *OTS1*) in wheat (*Triticum aestivum* L.) has led to improved plant growth under water stress conditions. Transformed wheat (pUBI-*OTS1*) displayed enhanced growth and delayed senescence under water deficit when compared with untransformed Gamtoos-R genotype or plants carrying an empty vector. Transformed pUBI-*OTS1* plants also maintained a high relative moisture content (RMC), had a higher photosynthesis rate, and also had a higher total chlorophyll content when compared to untransformed plants or plants carrying an empty vector. SUMOylation of total protein also increased in untransformed plants but not in the At*OTS1* transformed plants. Our results suggest that SUMO-proteases may influence an array of mechanisms in wheat to the advantage of the crop to be more tolerant to water stress caused by drought. This is the first report to elucidate SUMOylation effects in the hexaploid crop wheat (*T. aestivum* L.).

## Introduction

Water stress, due to periods of drought, is one of the most important abiotic stressors hampering productivity in agriculture crops. It occurs episodically in many regions is and, in some instances, it will be continuous with no indication of ending. Bread wheat (*Triticum aestivum* L.) is a high commodity crop cultivated in many countries accross a wide range of agroecological conditions and lack of water due to drought severely affects wheat productivity. Plants tolerate such water stress due to an array of biochemical reactions leading to phenotypic plasticity. Wheat, as with most cereals, will counter lack of water by several mechanisms such as the induction of reactive oxygen species-detoxifying agents, modification of photosynthesis rate, altering gene expression, re-allocation of proteins and their turn-over, ultimately affecting growth rate (Cruz de Carvalho, 2008; Ford et al., 2011; Bowne et al., 2012). Wheat will also shorten its lifespan to reproduce prior to water resources becoming totally depleted, a phenomenon known as drought escape or the ephemeral strategy (Shavrukov et al., 2017). Drought escape is often associated with stunted growth since the plant primarily channels all it nutrients and energy for seed production. However, the seed quality and yield are usually negatively affected, an undesirable agronomical attribute (Zampieri et al., 2017).

Regulation of protein post-translation modification (PTM) by Small Ubiquitin-like Modifiers (SUMO) is further an important biochemical mechanism to regulate plant growth during stress (Guerra et al., 2015). The process is referred to as SUMOylation, which is reminiscent of ubiquitination. The process employs its own set of analogous enzymes (E1, E2, and E3) to tag specific proteins through sequential catalysis activation, conjugation, and ligation, in respective reactions (Colby, 2006; Miura and Hasegawa, 2009; Hansen et al., 2017; Rytz et al., 2018). SUMOylation requires ATP and is a two-step reaction catalyzed by the heterodimeric E1 activating enzyme (SAE2/SAE1), forming a thioester bond between its catalytical cysteine and the C-terminal carboxyl group of SUMO. E2 conjugating enzyme (ubc9) receives this SUMO on the cysteine residue. With the aid of E3 SUMO ligase, SUMO is then carried to the ε-group of lysine of the target protein, forming an isopeptide bond (Desterro et al., 1999; Saracco et al., 2007; Gareau and Lima, 2010). SUMO conjugation of proteins leads to changes in their stability, availability, and functionality which could be detrimental to the plant (as reviewed by Botha et al., 2017). Liu et al. (2016) further revealed that the enzymes involved in the SUMOylation process deviated from conventional gene transcription by using a downstream gene *39-UTR*, making use of a functional Pol V-dependent pathway.

SUMOylation is also important during plant development. Engineered *Arabidopsis thaliana*, which had a mutation in either SAE1/2 encoding E1 or SCE1 encoding conjugating (E2) enzyme, suffered serious growth defects (Saracco et al., 2007). However, SUMOylation is a reversible process. SUMO proteases act as iso-peptidases that specifically cleave the SUMO–substrate linkage during a process called deSUMOylation that allows for the recycling of free SUMO and ensuring homeostatic control of SUMO-mediated signaling (Johnson, 2004; Capili and Lima, 2007; Conti et al., 2008). In recent years, much emphasis has been placed to understand the de-SUMOylation process and the function of cysteine proteases to unravel the process of SUMOylation. Seven SUMO cysteine proteases, belonging to the CE and CP clans (Botha et al., 2017) have been identified from *A. thaliana*. However, only proteases *AtULP1a (At3g06910*), OVERLY TOLERANT TO SALT-1 (*OTS1*) (*At1g60220*), OVERLY TOLERANT TO SALT-2 (*OTS2) (At1g10570*), and EARLY IN SHORT DAYS 4 (*AtESD4) (At4g15880*) have been functionally characterized (Reeves et al., 2002; Conti et al., 2008; Srivastava et al., 2016a,b).

The cysteine protease *OTS2* for example acts redundantly to modulate salt stress response but plants lacking SUMO proteases cannot endure salt stress (Conti et al., 2008, 2014). *A. thaliana* mutants expressing OTS1/2 are also more resistant to *Pseudomonas syringae* and have higher salicylic acid content (Bailey et al., 2016). *OTS1* overexpression in *A. thaliana* further increases salt tolerance with a concomitant reduction in SUMOylated protein production (Reeves et al., 2002; Conti et al., 2008). Srivastava et al. (2017) recently found that transgenic rice (*Oryza sativa* L. cv. *Nipponbare*) overexpressing *OTS1* (*OsOTS1*) were more salt-tolerant but also more drought-sensitive possibly due to a reduced amount of the drought responsive transcription factor OsbZIP23 leading to suppressed drought responsive gene expression. Overexpressed OsOTS1 SUMO protease thereby directly targets the ABA and drought responsive transcription factor OsbZIP23 for de-SUMOylation affecting its stability. In contrast, OsOTS-RNAi lines, with reduced OsOTS1 SUMO protease production, had an increased abundance of OsbZIP23 and increased drought responsive gene expression resulting in better drought tolerance in rice (Srivastava et al., 2017). However, besides the recent study in rice, there is still very little known about the function of *OTS1* in crops other than rice. Also, there is little known about any consequences of changed *OTS1* expression in other plants than rice or the non-crop plant Arabidopsis regarding any possible benefits to a plant to withstand water stress caused by drought. In general, exposure of a plant to water stress caused by drought induces variations in osmotic potential and affects cellular turgor pressure, causing vacuole disruptions, and an increased expression of cysteine proteases (Seki et al., 2002; Kidrič et al., 2014; Botha et al., 2017). In addition, oxidative stress greatly increases during water stress conditions leading to the formation of reactive oxygen radicals (hydrogen peroxide), a reduction in the availability of amino acids and ultimately protein degradation. In addition, the abundance of key photosynthetic proteins such as the chloroplast-located ribulose-1,5-bisphosphate carboxylase/oxygenase (RuBisCO) (Khanna-Chopra, 2011) is affected under drought resulting in the substantial reduction of photosynthesis (Chaves et al., 2008; Perdomo et al., 2017).

The purpose of this study was therefore to advance our understanding of the function of SUMO proteases in stress protection particular during water stress conditions. Our study was thereby aimed to specifically investigate if over-expression of cysteine protease OVERLY TOLERANT TO SALT-1 (At*OTS1*) from *A. thaliana* (ecotype Columbia-0) in bread wheat (*T. aestivum* L.) will provide improved tolerance to water stress, as the elucidation of SUMOylation has so far almost exclusively been conducted in the non-crop species *A. thaliana.* In selected transformed wheat plants we particularly measured plant growth as well as photosynthetic activity, changes in proteolytic and antioxidative enzyme activity, and amino acid production after exposure of plants to water stress caused by drought exposure when compared with untransformed plants and plants carrying an empty vector.

## Materials and Methods

### Plant Treatment and Plant Growth Analysis

For the assessment of the response of the plants overexpressing At*OTS1* to water stress, seeds of transformed plants overexpressing At*OTS1* (Gamtoos-R pUBI-*OTS1;* three third generation plants from independent transgenic events) (Le Roux, 2015; Botha-Oberholster et al., 2017), plants only transformed with an empty vector (empty-pUBI), and non-transformed wild type plants were germinated in pots containing an equal volume of sand and soil in a greenhouse at 20°C (16 h day:8 h night cycle). Pots were equipped with a dripper irrigation system containing nutrients (Multifeed TM, South Africa). Water supply was stopped when plants reached the final extension stage of growth (58–65 days after germination) corresponding to phase 45 of the Zadoks’ scale (Supplementary Figure S1; Zadoks et al., 1974; Vendruscolo et al., 2007) (day 0 in experiments), as heading and grain filling were shown to be one of the most sensitive growth stages during wheat development to apply drought stress (Ahmed et al., 2000; Ihsan et al., 2016). After stopping any further water supply, all untransformed plants or plants carrying an empty vector died after 7 days (no further measurements were made with these plants after 7 days), while the plants overexpressing At*OTS1* were analyzed until day 14 post watering (pw).

For plant growth analysis, plant height, and flag leaf length and width were measured according to Aase (1977) using a line gauge (unit of measurement in mm). In the case of plant height all the individual tillers of the plant were measured from the ground to the tip of the tallest tiller of the plant (*n* = 20). Plant fresh and dry weight, which included turgid weight, was determined to calculate the relative moisture content (RMC) (Sade et al., 2014, 2015). Soil samples (*n* = 3) to 150 mm depth were also collected and the soil wet mass was determined after drying the soil in an oven at 105°C for 48 h then the soil was weighed and the gravimetric soil moisture content determined (Black, 1965).

### Photosynthesis (F_v_/F_m_), Stomatal Conductance and Chlorophyll Determination

Stomatal conductance was measured at three positions on each leaf surface using a leaf porometer (model SC-1, Decagon Devices, Inc., Pullman, WA, United States) as previously described (Zarco-Tejada et al., 2000). Rate of photosynthesis was measured according to Strasser et al. (2004) making use of chlorophyll fluorescence (ChlF) induction transients (O-J-I-P), using a hand-held Chlorophyll Fluorometer (model: OS-30P; Manufacturer: Opti-Sciences, Inc., United States) QQ (Opti-Sciences, 2004). Dark adaptation clips were applied to leaves for 20 min (prior to reading) to achieve a flush out of assimilates. Technical repeats for both instruments were recorded at different places from the tip to the base of the flag leaf to represent the entire leaf surface. All measurements were taken at the onset of the water stress treatment (day 0) and then at days 7 and 14 pw.

Extraction of chlorophyll was done according to Arnon (1949) and spectrophotometrically determined with the SmartSpec^TM^ Plus BioRad. Chlorophyll content was calculated using the Arnon (1949) equation. All measurements were carried out with three biological repeats (*n* = 3) and conducted in triplicate (*n* = 9).

### Enzyme Activity and Protein Determination

Extraction of protein for enzyme activity analyses was performed as described in Botha et al. (2014). For enzymatic analysis, leaf tissue was snap-frozen in liquid N_2_ and then ground to powder, followed by the addition of 500 ml of ice-cold 100 mM potassium phosphate buffer (pH 7.5) containing 1 mM ethylenediaminetetraacetic acid (EDTA) and 1% (m/v) polyvinylpyrrolidone (PVP). After centrifugation (25,000 × *g* for 20 min at 4°C), the supernatant was used for enzyme assays. All enzyme activity measurements were conducted with three biological repeats (*n* = 3) and conducted in triplicate (*n* = 9).

Peroxidase activity was determined following a modified method of Zieslin and Ben-Zaken (1991) with 0.1 M sodium phosphate buffer (pH 5), 3 mM H_2_O_2_, 3 mM guaiacol, and an aliquot of the enzyme extract (Botha et al., 2014). The formation of tetraguaiacol was monitored at 470 nm. POX activity was expressed as mmol tetraguaiacol min^-1^ mg^-1^ protein.

Glutathione S-transferase (GST) enzyme activity was measured as described by Venisse et al. (2001) using 0.1 M phosphate buffer (pH 6.5), 3.6 mM reduced glutathione, 1 mM 1-chloro-2,4-dinitrobenzene (DNB), and an aliquot of the enzyme extract (Botha et al., 2014). The formation of GS-DNB conjugate was monitored at 340 nm. GST activity was expressed as mmol GSH min^-1^ mg^-1^ protein.

### Protein Concentration

Protein concentration in plant extracts was determined according to the method described by Bradford (1976) with the Bio-Rad protein assay reagent with bovine albumin (Bio-Rad Laboratories Inc., Hercules, CA, United States) as a standard, and using a plate reader (Glomax Spectrophotometer, Promega, Sunnyvale, CA, United States), as described by Rylatt and Parish (1982).

### SDS-PAGE and Western Blot Analyses

Total protein was isolated from plants according to Dehesh et al. (1986) with the addition of 2 mM phenylmethylsulfonyl fluoride (PMSF) to the extraction buffer. Protein concentration was determined as described above. Total protein (25 μg) dissolved in 4X Laemmli buffer (Bio-Rad, Hercules, CA, United States) was denatured at 95°C for 5 min and then separated on a Mini-Protein TGX gradient gel (4–15%, v/v) according to the manufacturer’s instructions (Bio-Rad, Hercules, CA, United States). Separated proteins were transferred to a polyvinylidene difluoride membrane (Hybond-P, Amersham Biosciences) with a Bio-Rad *Trans-*Blot^®^ SD semi-dry transfer cell apparatus according to the manufacturer’s instructions (Bio-Rad, Hercules, CA, United States). Membranes were blocked with 3% (m/v) bovine serum albumin (BSA) and probed with a polyclonal antibody against the large (LSU) and small (SSU) Rubisco Subunit (1:50,000) (Botha and Small, 1987) and a human anti-SUMO1 monoclonal antibody (1:2,500) (UBPBio, Aurora, United States) diluted in phosphate buffered saline (PBS) containing 3% (m/v) BSA. Protein detection was done with alkaline phosphatase conjugated Donkey Anti-Mouse (Abcam) (1:2,500) or goat anti-rabbit (1:7,000) (Sigma-Aldrich, St. Louis, MO, United States) antisera in conjunction with nitro blue tetrazolium and 5-bromo-4-chloro-3-indolyl phosphate (Sigma-Aldrich, St. Louis, MO, United States).

To quantify protein expression, membranes were digitized, and bands intensities were analyzed by densitometry using ImageJ software with a standard setting (ImageJ Software, NIH, Bethesda, MD, United States). Results were expressed as relative abundance, after normalization following two-way ANOVA.

### Protease Determination

Extraction of total proteases was carried out with 0.1 M citrate-phosphate buffer (CP, pH 5.6) containing 10 mM L-cysteine. For extraction, leaf tissue was ground to a powder using liquid N_2_, then cold CP buffer (20 mM, pH 5.6) was added. After centrifugation (25,000 × *g* for 20 min at 4°C), the protease containing supernatant was analyzed on a gradient acrylamide gel (5–15%). For gel preparation, the Hoefer^TM^ SG Series Gradient Makers system was applied. Solution one (S1) contained a 30% acrylamide:*N*,*N*′-methylenebisacrylamide (29:1) solution, 1% (m/v) sodium dodecyl sulfate (SDS), 0.2% (m/v) gelatine, 10 % (v/v) tetramethylethylenediamine (TEMED), ammonium persulfate (APS, 10%, m/v), and 1.5 M Tris pH 8.8. The second solution (S2) contained all ingredients as in S1 with the exception of gelatine and 30% acrylamide solution (equates to highest %T). Gels were pre-electrophoresed at 50 V for 60 min in the gel buffer storage condition at 4°C, and then 80 mg of protein sample, with or without addition of the cysteine proteinase inhibitor E64 (Barrett et al., 1982; Matsumoto et al., 1999), was loaded and proteins were separated at 15 mA for 2 h. After separation, the gels were carefully removed from the glass plates and washed three-times in a renaturing buffer (2.5% v/v Triton-X 100 and 5 mM cysteine) and subsequently incubated in developing buffer (0.5% v/v Triton-X 100, 50 mM Tris–HCl, pH 7.5 and 5 mM CaCl_2_, 1 mM ZnCl_2_, 10 mM cysteine) for 24 h. The gels were stained with Coomassie R-250 and destained until clear zones, which indicate protease activity due to gelatine degradation, were visible against the dark blue background (Palma et al., 2002).

### Amino Acid Extraction and Quantification

Leaf material was dried at 60°C for 24 h. Samples were then finely ground to powder to which 0.5 ml of 6 M HCI containing norleucine (250 ppm) as an internal standard was added. Amino acids analysis was conducted using the AccQ•Tag^TM^ Ultra Derivatization Kit (Waters, United States) following the manufacturer’s instructions, and as described by Boogers et al. (2008). A photo diode array detector was used to detect the derivatized amino acids at 260 nm. Amino acids were identified by co-elution with amino acid standard H (Pierce, United States) as well as commercially available individual amino acids (Sigma, United States).

### Data Analysis and Statistics

All measurements were made with three biological repeats (*n* = 3) with measurements done in triplicate (*n* = 9). Mean values are presented with their standard deviation (SD) and analyzed using Graphpad Prism software version 5.0 (Motulsky, 2014^1^). Statistical validation and significance (*p* = 0.05) was determined with one-way analysis of variance followed by post-*t* Dunnett’s test.

## Results

### Phenotypic Response to Water Stress

**FIGURE 1:**
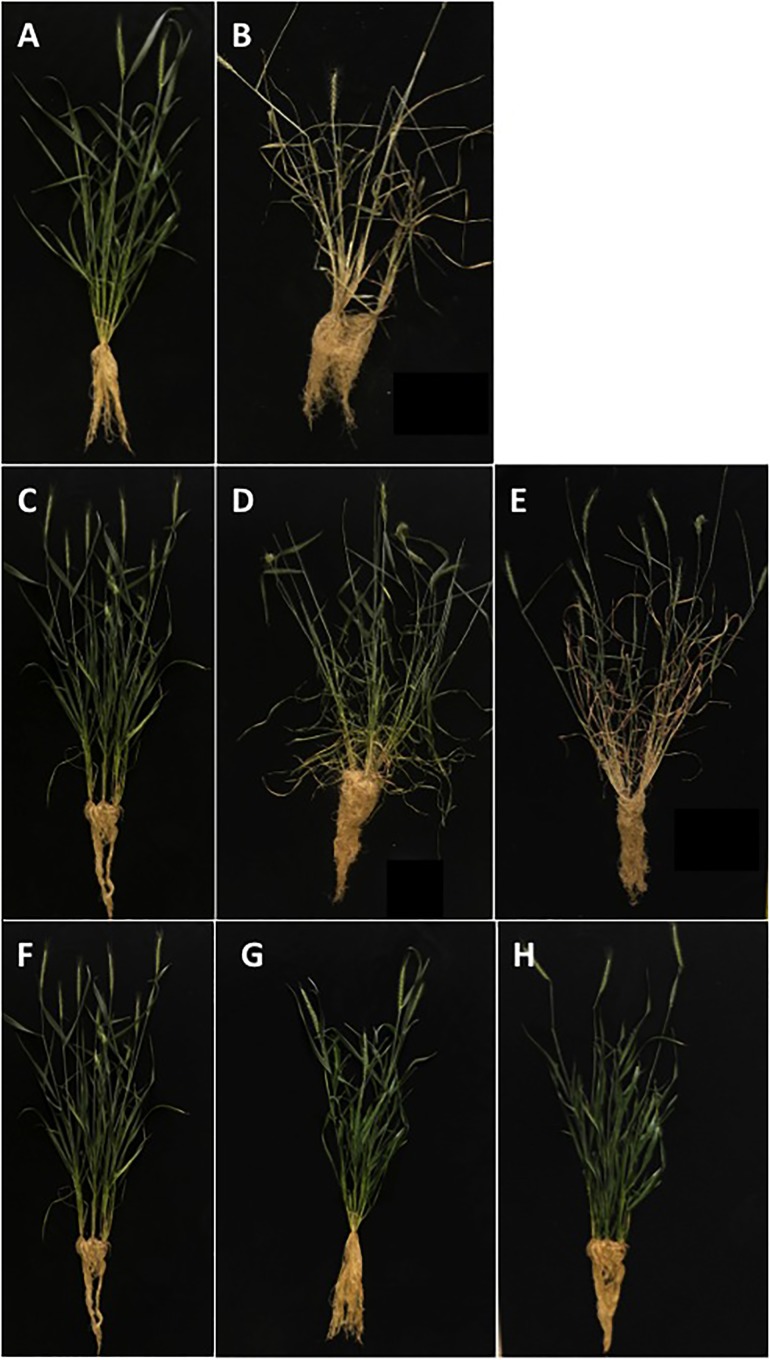
Phenotypic response of untransformed (WT Gamtoos-R) and transformed wheat (pUBI-OTS1) prior to **(A,B)**, and after induction of water stress **(C–E)**. Where **(A,B)** is untransformed wheat at day 0 **(A)**, and day 7 **(B)**, and **(C–E)** is transformed wheat at day 0 **(C)**, day 7 **(D)**, and day 14 **(E)**. Also depicted are the phenotypes of transformed wheat plants (pUBI-*OTS1*, T1; pUBI-*OTS1*, T2; pUBI-*OTS1*, T3) **(F–H)**.

To assess the responses of either untransformed plants or transformed plants containing an empty-pUBI, or transformed plants containing At*OTS1* (pUBI-*OTS1*), the plants were grown in the greenhouse and phenotypically assessed at day 53 before exposure to water stress. Figure 1 illustrates the phenotype of untransformed (A–B) and transformed wheat plants expressing At*OTS1* (C–E) before and after exposure to water stress. The different types of plants had no significant difference in leaf length or width (Table 1). However, plant height of transformed pUBI-*OTS1* plants (average 710 mm) differed significantly (*p* = 0.005, *n* = 20) from that of the untransformed wild type plants (400 mm) (Table 1). When water stress was imposed, the untransformed plants were wilted and started to senesce after 2 days. By day 5, untransformed plants and plants transformed with the empty-pUBI were severely wilted and displayed symptoms of chlorosis, bleaching, and leaf curling, and by day 7, these plants were dead (Figure 1B). In contrast, the transformed pUBI-*OTS1* plants only expressed similar symptoms after 14 days of exposure to water stress (Figure 1E).

**Table 1:** Plant height, as well as flag leaf length and width of untransformed (WT Gamtoos-R) and wheat transformed with pUBI-*OTS1* or empty vector (pUBI) prior to exposure to water stress.

Genotype	Plant organ		
	
	Plant height	Leaf length	Leaf width
	(mm)	(mm)	(mm)
WT Gamtoos-R	400 ± 8.0a	380 ± 5.0a	65 ± 5.0a
Empty-pUBI	410 ± 18.0b	360 ± 18.0a	66 ± 5.0a
Transgenic pUBI-*OTS1*	710 ± 55.0c	370 ± 25.0a	82 ± 9.0a


*Three independent transgenic lines were measured, with each one being compared with the non-transgenic control plant (WT Gamtoos-R). Experiments were repeated three times. Error bars indicate SD (*n* = 9) and significance was set at *p* = 0.05.*

**FIGURE 2:**
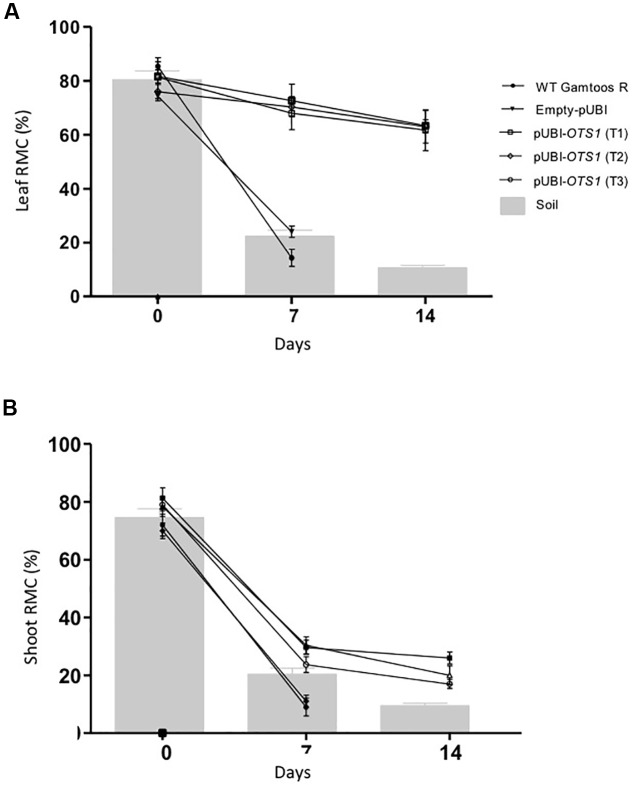
Comparative analysis of relative moisture content (RMC) measured in the leaves **(A)**, and shoots **(B)** prior to and after induction of water stress. The gravimetric readings of the soil are superimposed against the RMC. Indicated are the RMC of untransformed (WT Gamtoos-R) and wheat transformed with pUBI-*OTS1* or empty vector (pUBI), emphasizing how they maintain RMC under deficient soil moisture. Error bars indicate SD (*n* = 9) and significance was set at *p* = 0.05.

To study the level of water loss experienced by the different types of plants, relative moisture content (RMC) of leaves and shoots, which in turn coincides with the gravimetric analysis of soil water content, was also assessed (Figure 2). Transformed pUBI-*OTS1* plants had a significant higher amount of water in its leaves after exposure to water stress when compared to untransformed and empty-pUBI plants (*p* = 0.05). Although both groups initially had an RMC of ± 80% at a soil moisture content of about 80%, RMC in both leaves and shoots greatly declined in the untransformed plants after 7 days of water stress exposure and leaves had only a RMC of 15%. In contrast, pUBI-*OTS1* transformed plants still had a much higher leaf RMC (± 75%) 7 days after drought exposure. RMC further declined but only to 60% RMC 14 days after induced water stress exposure (Figure 2). A similar different response to induced water stress was found when the shoot RMC was measured for the different types of plants. Again, transformed pUBI-*OTS1* plants maintained a higher RMC after exposure to water stress (7 or 14 days pw) when compared to untransformed plants or plants transformed with an empty vector (Figure 2).

### Chlorophyll, Photosynthesis and Stomatal Conductance

**FIGURE 3:**
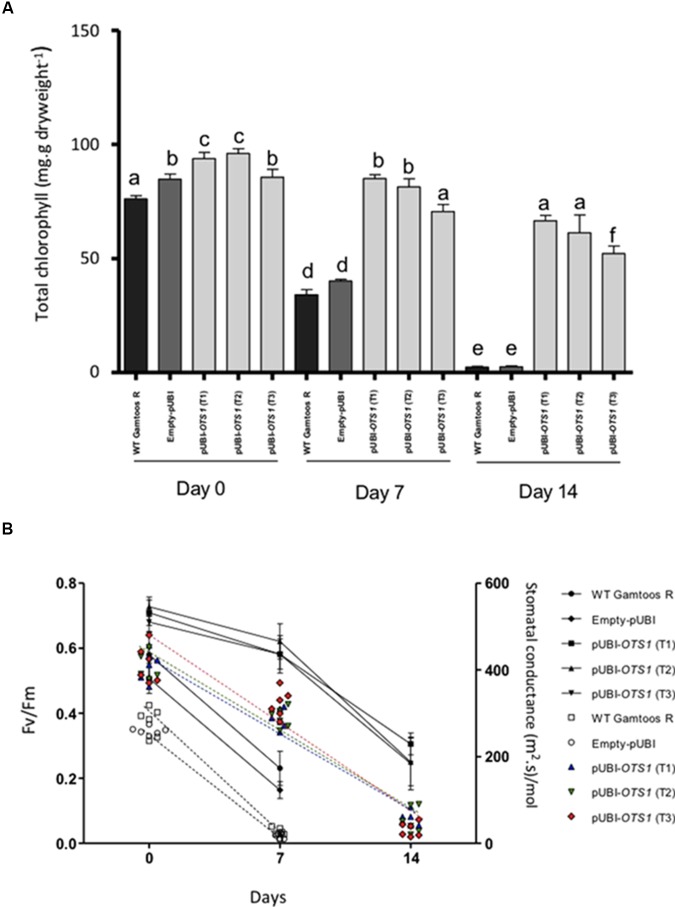
**(A)** Total chlorophyll measured in the untransformed (WT Gamtoos-R) and wheat transformed with pUBI-OTS1 or empty vector (pUBI), prior to (day 0) and after exposure to water stress (day 7, and 14). Error bars indicate SD (*n* = 3). Similar letters on the bars indicate no significant difference, whereas different letters indicate significance at *p* = 0.05. **(B)** Rate of photosynthesis (Fv/Fm) (line graph) and stomatal conductivity (scatter plot) prior to (day 0) and after exposure to water stress (day 7, and 14). Photosynthesis significance was determined by *p* = 0.005 where *n* = 6 and error bar indicate SD.

We then measured chlorophyll content, photosynthesis rate and stomatal conductance in the untransformed and transformed plants (Figure 3). Chlorophyll content was significantly reduced (*p* ≤ 0.05) after exposure of plants to induced water stress (Figure 3A). However, the transformed pUBI-*OTS1* plants had consistently higher chlorophyll content when compared with untransformed and empty-pUBI plants. A significant reduction in chlorophyll content was only observed in the pUBI-*OTS1* transformed plants 14 days after exposure to water stress, while untransformed and empty-pUBI plants had significantly less (*p* = 0.05) chlorophyll already 7 days after exposure to water stress.

A significant difference in the photosynthesis rate was also measured in the plants. pUBI-*OTS1* transformed plants had a higher (∼15%) rate than untransformed and empty-pUBI plants. After exposure to induced water stress, the photosynthetic rate declined but pUBI-*OTS1* transformed plants always had the highest rate when compared to untransformed or empty-pUBI plants (Figure 3B).

Stomatal conductivity measured in the untransformed and transformed plants also declined significantly with the onset of water stress (*p* = 0.05) as indicated by the scatter plot (Figure 3B).

### Change in Amino Acid Composition

We then characterized the amino acid composition following water stress treatment in the plants. Before exposure to water stress, transformed pUBI-*OTS1* plants had higher levels of most of the measured free amino acids, except for methionine and phenylalanine which were higher in untransformed plants (Table 2).

**Table 2:** Levels of free amino acids in leave material of WT Gamtoos-R and transgenic PUBI-*OTS1* measured prior to (day 0) and after induction of water stress (day 7 and 14).

Genotype	Days	Free amino acid content [Concentration in % (m/m) dry solid]															
		
		his	ser	arg	gly	asp	glu	thr	ala	pro	lys	tyr	met	val	lle	leu	phe
**WT Gamtoos-R**	**0**	0.1	0.31	0.21	0.3	0.5	0.49	0.24	0.39	0.28	0.32	0.225	0.61	0.28	0.15	0.28	0.84
	**7**	0.11	0.2	0	0.28	0	0.55	0.3	0.4	0.88	0.5	0.14	2.51	0.15	0.32	0.6	1.65
**pUBI-*OTS1***	**0**	0.11	0.325	0.32	0.31	0.56	0.596	0.24	0.396	0.295	0.395	0.21	0.54	0.31	0.19	0.41	0.32
	**7**	0.11	0.314	0.36	0.3	0.57	0.75	0.25	0.45	0.4	0.4	0.22	0.62	0.23	0.21	0.9	0.6
	**14**	0.12	0.298	0.296	0.26	0.56	0.6	0.15	0.33	0.56	0.31	0.19	0.52	0.26	0.21	0.225	0.595


*Measurements was only taken for the WT Gamtoos-R until day 7, as it suffered irreversible damage and was dead by day 7. Red = increases > 50% from day 0. Green = decreased > 50% from day 0.*

After exposure to water stress for 7 days, transformed plants overexpressing At*OTS1* still had more free amino acids when compared with untransformed plants with the exception of proline, lysine, methionine, isoleucine, and phenylalanine, which were at higher levels in the untransformed plants. The levels of the free hydrophobic amino acids methionine, proline, and phenylalanine more than doubled after induction of water stress in both transformed and untransformed plants. Interestingly, the arginine and aspartate content was undetectable after 7 days pw in the untransformed plant, while these remained unchanged in the transformed plants overexpressing At*OTS1*. After 14 days pw a notable decrease in leucine levels are observed (Table 2).

### RuBisCO and SUMO Expression

**FIGURE 4:**
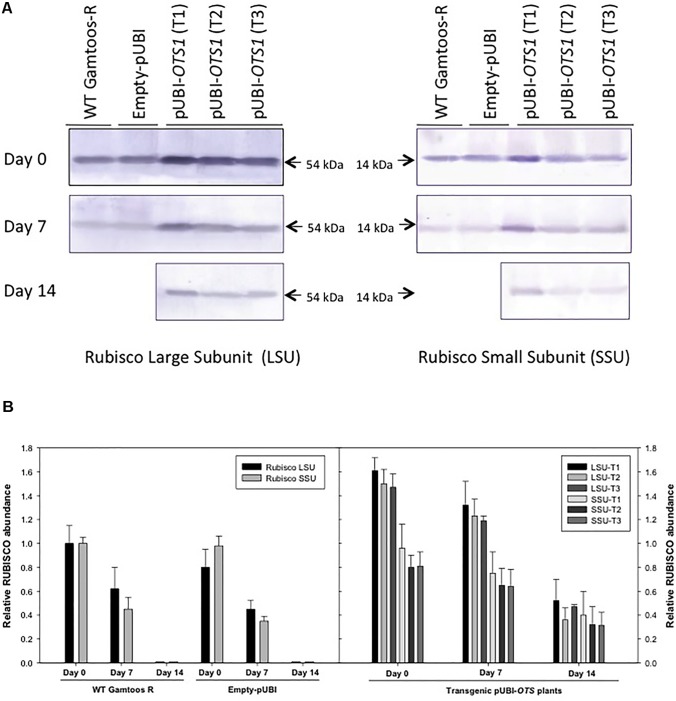
**(A)** Protein blot of crude extract from untransformed (WT Gamtoos-R) and wheat transformed with pUBI-*OTS1* or empty vector (pUBI) prior to (day 0) and after exposure (day 7 and 14) to water stress, probed with anti-Rubisco (LSU) and (SSU) IgG. All lanes were loaded with 20 μg total protein. Blots were probed with a 1:7 000 dilution of the polyclonal IgG against LSU, SSU. The leaf proteins were resolved by 12% (w/v) sodium dodecyl sulfate–polyacrylamide gel electrophoresis prior to transferring to nitrocellulose. Images were cropped for presentation purposes. T1–T3 represents three independent transgenic events. **(B)** Gel densitometric analysis of the protein blot in **(A)** of the rubisco large (LSU, 54 kDa) and small (SSU, 14 kDa) subunits in the leaf crude protein extracts from untransformed (WT Gamtoos-R) and wheat transformed with pUBI-*OTS1* or empty vector (pUBI) prior to (day 0) and after exposure (day 7 and 14) to water stress. Data are expressed as relative levels of rubisco protein compared with the basic level in control line (mean value of 1.0). Each bar is the mean of three independent values (biological replicates) ± SE.

To further study the effect of water stress, particularly on RuBisCO and SUMO expression in untransformed, empty vector transformed and the pUBI-*OTS1* transformed plants, protein extracts were analyzed by SDS-PAGE and protein blots were probed with RuBisCO and SUMO1 antibodies (Figure 4). Protein blots probed with anti-LSU (RuBisCO large subunit) and anti-SSU (RuBisCO small subunit) IgGs revealed two cross-reacting peptides with sizes of 56 ± 4 kDa (LSU) and 15 ± 2 kDa (SSU) respectively, which are the correct sizes for the two subunits in wheat (Botha and Small, 1987). To further estimate the relative abundance of the two subunits, the protein blots were scanned with a laser densitometer. Densitometric analyses of the blots revealed that LSU was always more abundant in the transformed pUBI-*OTS1* plants than in the untransformed plants or empty-pUBI plants before and also after 7 day of induced water stress treatment (Figure 4). Both subunits decreased in abundance in the untransformed and empty-pUBI plants when water stressed. In contrast, the abundance of the two subunits only significantly decreased in the transformed pUBI-*OTS1* plants when plants were exposed to water stress for 14 days (Figure 4A).

**FIGURE 5:**
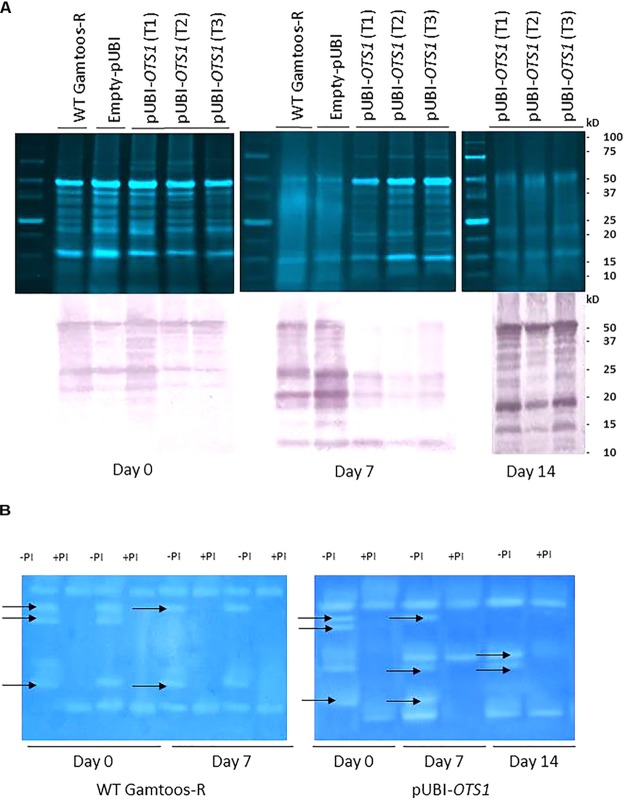
**(A)** **Top:** Crude protein separated on a 12.5% SDS-PAGE from untransformed (WT Gamtoos-R) and transformed pUBI-*OTS1* wheat prior to (day 0) and after exposure to water stress (day 7 and 14). All lanes were loaded with 20 μg total protein. **Bottom:** Untransformed (WT Gamtoos-R) and wheat transformed with pUBI-*OTS1* or empty vector (pUBI) prior to (day 0) and after exposure (day 7 and 14) to water stress, probed with anti-SUMO IgG. All lanes were loaded with 20 μg total protein. Blots were probed with a dilution of 1:10 000 dilution of monoclonal IgG against SUMO1. Images were cropped for presentation purposes. T1–T3 represents three independent transgenic events. **(B)** Gradient Zymograms depicting proteolytic activity of untransformed and transgenic pUBI-*OTS1* prior to (day 0) and after exposure to water stress (day 7 and 14). Zymograms (gradient 5–15%) were casted and in all cases 35 μg protein was loaded. Inclusion of an incubation step with 0.1 mM Cystein Protease inhibitor (E-64) performed at pH 7, enabled for the identification of cysteine proteases. Lanes with +PI refers to treatment with protease inhibitor; whereas –PI refers to no inhibitor treatment. Arrows indicated bands that were removed after treatment with the 0.1 mM Cystein Protease inhibitor (E-64). The presented data is representative of two independent experiments. Images were cropped for presentation purposes, and the contrast was adjusted (10%).

To study the changes in SUMO cysteine proteases, blots of separated crude protein extracts were also probed with monoclonal anti-SUMO1 IgG. Several cross-reacting peptides were found ranging in sizes from 50 ± 10 kDa to 10 ± 5 kDa but with no difference in the profile between the cross-reacting SUMO1 peptides in the untransformed and transformed plants overexpressing *OTS1* (Figure 5A). However, several cross-reacting SUMO1 peptides present in the profile of untransformed plants, was absent in the transformed plants overexpressing At*OTS1* after exposure to water stress.

To further elucidate whether the peptides on the protein blots were cysteine proteases, a protease inhibitor E64 specific to cysteine proteases, were included in the protein analysis before proteins were separated on gradient zymograms (Figure 5B and Supplementary Figure S2). A comparison between the protein profiles of untransformed and transformed pUBI-*OTS1* plants before exposure to induced water stress showed no difference in the profiles of proteases in the two types of plants, with each having five protein bands with proteolytic activity. Addition of the cysteine protease inhibitor E64 blocked the activity of three proteases. After 7 days of exposure to water stress, the profile of transformed plants had two additional activity bands, the latter being an E-64 inhibitible cysteine protease not present in the proteolytic profile of the untransformed plants (Figure 5B). At day 14 pw, two bands were confirmed to be cysteine proteases in the transgenic pUBI-*OTS1* after inhibition with E-64.

### Enzyme Activity in Water Stressed Plants

**FIGURE 6:**
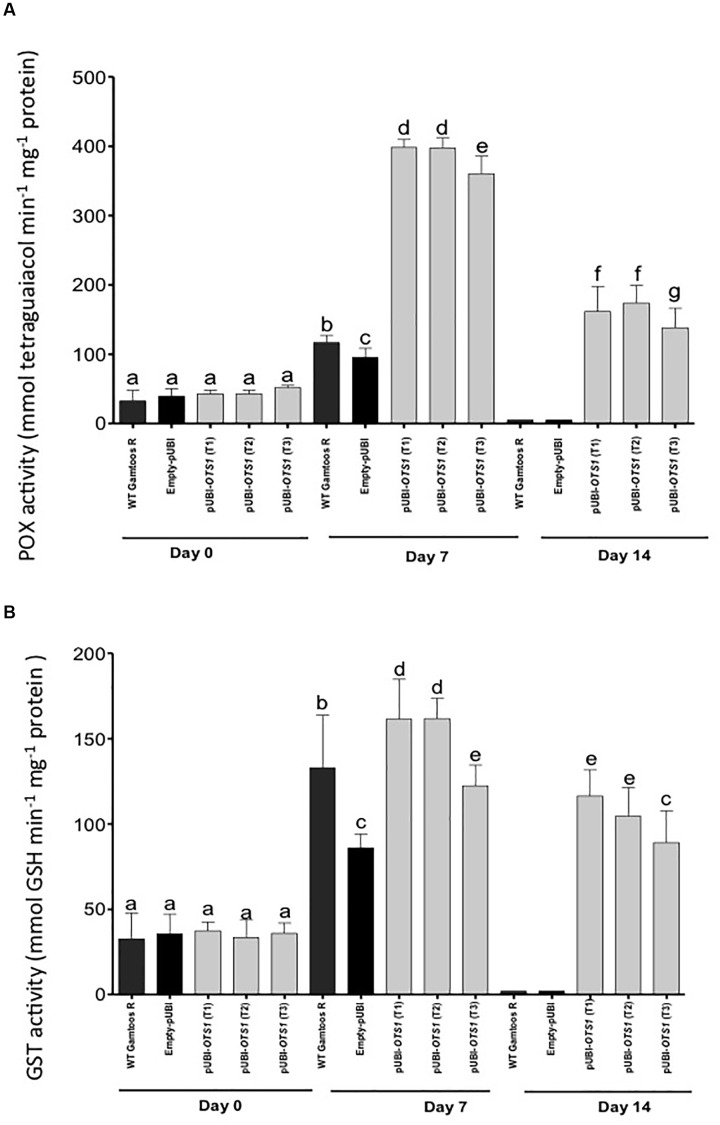
Changes in the peroxidase (POX) **(A)** and glutathione-S-transferase **(B)** activities measured in untransformed (WT Gamtoos-R) and wheat transformed with pUBI-*OTS1* wheat prior to (day 0), and after induction of water stress (day 7 and 14). POX activity was measured by the formation of tetraguaiacol monitored at 470 nm, while GST activity represents the formation of GS-DNB conjugate at 340 nm. Error bars indicate SD (*n* = 3). Bars with the same letters indicate no significant difference, whereas different letters indicate significance at *p* = 0.05.

Finally, we also measured the activity of enzymes usually associated with plant stress and stress induced oxygen species. POX activity differed significantly between the pUBI-*OTS1* and the untransformed and empty-pUBI plants (*p* = 0.05) (Figure 6). After exposure to water stress, POX and GST activity greatly increased in transformed pUBI-*OTS1* plants, but not in the other types of plants.

## Discussion

Overexpression of At*OTS1* changed in our study the phenotype of wheat plants with plants becoming taller and having broader leaves. Overexpression of At*OTS1* further provided better tolerance to drought by delaying the onset of wilting observed in the untransformed plants. Although water stress significantly decreased the RMC in untransformed plants, such dramatic decrease was not found in the At*OTS1* overexpressing transformed plants. RMC generally serves as an essential indicator as to how the plant manages its water stress condition, which is directly related to soil water content (Hammad and Ali, 2014). Despite a massive 60% decline in soil water content, the transformed pUBI-*OTS1* plants maintained a high RMC. It was previously shown that overexpression of an array of genes provide drought tolerance (Lawlor, 2012 and references within) by maintaining full turgor pressure in cells during water stress conditions.

Our results also directly contradict the recent findings reported by Srivastava et al. (2017). Although a slight increase in shoot length in *OsOTS1* overexpressing rice plants under non-stressed conditions was also found, *OsOTS1* overexpression increased, in contrast to our findings, the sensitivity of rice plants against induced water stress with *OsOTS1* overexpressing plants also having a reduced amount of the drought responsive transcription factor *OsbZIP23* leading to suppressed drought responsive gene expression. In contrast, silencing of *OsOTS1* expression increased the adaptability to drought conditions. Arguably, the contradicting findings might be due to differences in rice and wheat drought sensitivity. In addition, the possible action of *OTS1* in these plant systems during stress response processes cleaving the SUMO–substrate linkage during deSUMOylation might be very different. Generally, rice is more sensitive to drought and wheat can also better adapt to drought conditions via high osmotic adjustment and recovery after stress (Daryanto et al., 2017). In a recent meta-analysis study, Zhang et al. (2018) also found that drought decreased agronomic traits differently between wheat and rice among different growth stages. In addition, a further reason for our contradicting finding might be the application of a heterologous system using an Arabidopsis-derived *OTS1* gene expressed in wheat while Srivastava et al. (2017) overexpressed a rice *OTS1* in a homologous rice system which could have caused gene silencing and hence increased drought sensitivity.

In this study, we also found that delaying the onset of induced water stress in At*OTS1* transformed plants had several beneficial consequences for survival following induced water stress. These plants maintained a higher content of chlorophyll across the period of induced water stress, when compared to untransformed plants. Drought induce a reduction of total chlorophyll content as already found in various crops (Mafakheri et al., 2010; Gholaminr and Khayatnezhad, 2011; Hailemichael et al., 2016). However, after long exposure to water stress (14 days with a final soil water content ± 15%), the At*OTS1* transformed plants also developed wilting symptoms that were comparable to the symptoms developed in the other plants 7 days after water stress exposure. A prolonged water stress is generally associated with destruction or disorganization of thylakoid membranes, subsequently decreasing and depleting chlorophyll and its synthesis which is followed by plant death (Nilsen and Orcutt, 1996; Montagu and Woo, 1999).

The At*OTS1* transformed plants had further a higher photosynthetic rate when compared to untransformed plants (Marcus et al., 2008). The photosynthetic capacity (Fv/Fm ratio) of transformed plants remained functional longer than that of the other plants despite exposure to water stress. Many photosynthetic proteins (photosystem I and II subunits; CAB-binding proteins 2, 3, and CP29; protein import receptors; GTP-binding proteins; ferredoxins; ADP/ATP translocase) are substrates for SUMOylation (Elrouby and Coupland, 2010), thereby promoting dysfunctionality amongst these proteins. However, by overexpressing a SUMO-protease some of these proteins might have been more deSUMOylated and therefore more functional under drought-induced water stress.

Rate of photosynthesis is further characterized by the maximum quantum yield of the primary photochemical reaction in dark-adapted leaves (Parkhill et al., 2001). This provides an indication of the presence of photo-inhibition during water stress anticipation (Paknejad et al., 2007). A comparison of the Fv/Fm of untransformed and At*OTS1* transformed plants implied differential adaptive photosynthetic and photo-protective mechanisms after exposure to water stress. Since Fv/Fm ratio is collectively indicative of the Photosystem (PS) II functionality (Parkhill et al., 2001), our data suggest a unique preservation of PSII in transformed At*OTS1* overexpressing plants, which resulted in a slower decline in the ratio Fv/Fm under water stress.

Transformed pUBI-*OTS1* plants also had a higher abundance of RuBisCO when compared to untransformed plants under non-stress conditions. The RuBisCo abundance remained higher in At*OTS1* transformed plants even after exposure to water stress and only declined after long exposure to water stress (14 days after water stress exposure with a soil water content ±15%). RuBisCO consists of eight nuclear-encoded large subunits (RuBisCO LSU) and eight chloroplast-encoded RuBisCO small subunits (SSU) (Botha and Small, 1987). The large subunit contains the active site, while the small subunits are responsible for regulating the function and structure of RuBisCO (Raunser et al., 2009). RuBisCO protein accumulation is generally affected under stress conditions (Feller et al., 2008; Demirevska et al., 2009). After exposure to induced water stress, the abundance of both large and small subunits remained high in the At*OTS1* transformed plants with the large and small subunits declining only after long water stress exposure (day 14 after water stress exposure with a soil water content ± 15%). Since RuBisCO subunits interact not only with each other, but also require other protein partners for proper assembly and functioning (Raunser et al., 2009), increased deSUMOylation due to At*OTS1* overexpression might have protected in transformed plants the stability of the RuBisCO subunits during water stress.

We also found more proteolytic activity in transformed plants overexpressing At*OTS1* with more, and also more intense, activity bands particularly after exposure to induced water stress. Some of these protease activities could be blocked by the cysteine protease inhibitor E64 (Barrett et al., 1982; Matsumoto et al., 1999) particularly after 7 days of water stress exposure. However, we have so far not investigated if specifically any SUMO proteases are represented in these activity bands. In our protein expression analysis we also found as a consequence of At*OTS1* overexpression fewer cross reacting SUMO1 peptides. This finding possibly suggests that SUMOylation was lower in the At*OTS1* transformed plants which would be consistent with a previous finding in *A. thaliana* (Conti et al., 2008) and rice (Srivastava et al., 2016b). In *A. thaliana* for example, SUMOylation is directly influenced by the extent of stress exposure (Verma et al., 2018). Prolonged exposure to water stress leads to SUMO1/2 conjugation on a vast amount of proteins, influencing overall protein trafficking and its turnover. SUMOylation has the potential to change protein function or cause complete inhibition of function, therefore designated drought-associated proteins can no longer execute their function (Verma et al., 2018). Therefore, deSUMOylation is crucial for the survival of the plant during stress. Previously also suggested has been that there is a link between SUMO1/2 SUMOylation and drought tolerance through *OTS1* (cysteine protease) deSUMOylation activity conditions (Conti et al., 2008; Srivastava et al., 2016b; Verma et al., 2018).

The observed enhanced tolerance to drought in the At*OTS1* transformed plants can in part also be attributed to the up-regulation of the antioxidative system (Foyer et al., 1994). The cellular antioxidant system might be influenced by *OTS1*, since POX and dismutases possess predicted SUMO attachment sites (Srivastava et al., 2016b). It is therefore likely that SUMO conjugation occurs on these enzymes during stress, thereby affecting their activity and stability. GST activity also increased significantly after exposure to drought. GST essentially affords protein protection to various proteins under stress by detoxifying endogenous plant toxins that accumulate as a consequence of increased oxidative stress (Marrs, 1996). The observed GST increase during water stress would assure protein functionality by reducing oxidative damage (Cummins et al., 1999; Edwards et al., 2000; Roxas et al., 2000).

Finally, oxidative stress is often also associated with pronounced changes in amino acid amounts. Indeed, the observed increase in GST and POX activity in the untransformed (day 7) and At*OTS1* transformed plants (day 14) coincided with higher proline content, suggesting that proline may participate in scavenging reactive oxygen species in addition to its role as an osmolyte as previously reported in salt stressed plants (Hoque et al., 2007; Hossain et al., 2011; de Carvalho et al., 2013; Rejeb et al., 2014). Particularly interesting was the lower increase of the amino acid proline observed in the untransformed plants. Proline provides osmoprotection and the amount increases in many plant species, including maize, wheat and pea, following exposure to water stress (Rampino et al., 2006; Charlton et al., 2008; Witt et al., 2012). Since proline increased more rapidly in our untransformed plants due to water stress, At*OTS1* overexpression likely delays the natural response of production of an osmoregulant like proline as a consequence of drought-induced water stress.

In conclusion, we have found as new results that overexpression of At*OTS1* in wheat increased both shoot and leaf growth and lowered the abundance of SUMOylated proteins. In addition, we could demonstrate for the first time that At*OTS1* overexpression in wheat, in contrast to the *OsOTS1* rice, provides better tolerance to drought by delaying the onset of water stress. At*OTS1* overexpression maintained for longer vital cellular processes, such as photosynthesis, increased deSUMOylation thereby protecting the stability of the RuBisCO subunits during induced water stress. In addition, the upregulated antioxidative system activity lowered the stress response of an increase in the osmoregulatant proline. Lastly, as the SUMO proteases represented in our activity bands were not studied in detail, we need to study them in future to fully understand their role in the deSUMOlyation process that is responsible for delaying senesce in the At*OTS1* overexpressing plants during drought.

## Author Contributions

MlR and A-MB planned the study. MlR conducted the research and wrote the first draft. CvdV assisted with the plant transformation experiments. A-MB assisted with the protein assays. A-MB, KK, CvdV, and CC contributed to the interpretation of data and made editorial inputs.

## Conflict of Interest Statement

The authors declare that the research was conducted in the absence of any commercial or financial relationships that could be construed as a potential conflict of interest.

## Footnotes

1 http://www.graphpad.com/scientific-software/prism/

## References

[B1] Aase J. K. (1977). Relationship between leaf area and dry matter in winter wheat. *Agron. J. Abst.* 70 563–565. 10.2134/agronj1978.00021962007000040011x

[B2] Ahmed T. A. Tsujimoto H. Sasakuma T. (2000). QTLs associated with plant height and related characters in haploid wheat. *Breed. Sci.* 50 267–273. 10.1270/jsbbs.50.267

[B3] Arnon D. I. (1949). Copper enzymes in isolated chloroplasts, polyphenoxidase in *Beta vulgaris*. *Plant Physiol.* 24 1–15. 10.1104/pp.24.1.1 16654194 PMC437905

[B4] Bailey M. Srivastava A. Conti L. Nelis S. Zhang C. Florence H. (2016). Stability of small ubiquitin-like modifier (SUMO) proteases overly tolerant to salt1 and -2 modulates salicylic acid signalling and SUMO1/2 conjugation in *Arabidopsis thaliana*. *J. Exp. Bot.* 67 353–363. 10.1093/jxb/erv468 26494731 PMC4682439

[B5] Barrett A. Kembhavi A. Brown M. Kirschke H. Knight C. Tamai M. (1982). L-trans-epoxysuccinyl-leucylamido(4-guanidino) butane (E-64) and its analogues as inhibitors of cysteine proteinases including cathepsins B. H and L. *Biochem. J.* 201 189–198. 10.1042/bj2010189 7044372 PMC1163625

[B6] Black C. A. (1965). *Methods of Soil Analysis: Part I Physical and Mineralogical Properties.* Madison, WI: American Society of Agronomy.

[B7] Boogers I. Plugge W. Stokkermans Y. Q. Duchateau A. L. L. (2008). Ultra-performance liquid chromatographic analysis of amino acids in protein hydrolysates using an automated pre-column derivatisation method. *J. Chromatograp. A* 2 406–409. 10.1016/j.chroma.2007.11.052 18070624

[B8] Botha A.-M. Kunert K. Cullis C. (2017). Cysteine proteases and wheat (*Triticum aestivum* L) under drought: a still greatly unexplored association. *Plant Cell Environ.* 40 1679–1690. 10.1111/pce.12998 28664627

[B9] Botha A.-M. Van Eck L. Burger N. F. V. Swanevelder Z. H. (2014). Near-isogenic lines of *Triticum aestivum* with distinct modes of resistance exhibit dissimilar transcriptional regulation during *Diuraphis noxia* feeding. *Biol. Open* 3 1116–1126. 10.1242/bio.201410280 25361582 PMC4232770

[B10] Botha F. C. Small J. G. C. (1987). Control of ribulose 1,5-bisphosphate carboxylase synthesis in the cotyledons of *Citrullus lanatus*. *Plant Sci.* 53 121–129. 10.1016/0168-9452(87)90121-X

[B11] Botha-Oberholster A.-M. Van de Vyver C. Le Roux M. L. (2017). *Method of Enhancing Stress Tolerance of Monocotyledonous Plants.* Brazil Patent Application No. p3053za00scnc/U.S Patent Application No. Pct/ib2016/053936.

[B12] Bowne J. Erwin T. Juttner J. Schnurbusch T. Langridge P. Bacic A. (2012). Drought responses of leaf tissues from wheat cultivars of differing drought tolerance at the metabolite level. *Mol. Plant* 5 418–429. 10.1093/mp/ssr114 22207720

[B13] Bradford M. (1976). A rapid and sensitive method for the quantitation of microgram quantities of protein utilizing the principle of protein-dye binding. *Anal. Biochem.* 72 248–254. 10.1016/0003-2697(76)90527-3 942051

[B14] Capili A. Lima C. (2007). Taking it step by step: mechanistic insights from structural studies of ubiquitin/ubiquitin-like protein modification pathways. *Curr. Opin. Struct. Biol.* 17 726–735. 10.1016/j.sbi.2007.08.018 17919899 PMC2174906

[B15] Charlton A. Donarski J. Harrison M. Jones S. Godward J. Oehlschlager S. (2008). Responses of the pea (*Pisum sativum* L.) leaf metabolome to drought stress assessed by nuclear magnetic resonance spectroscopy. *Metabolomics* 4 312–327. 10.1007/s11306-008-0128-0

[B16] Chaves M. Flexas J. Pinheiro C. (2008). Photosynthesis under drought and salt stress: regulation mechanisms from whole plant to cell. *Ann. Bot.* 103 551–560. 10.1093/aob/mcn125 18662937 PMC2707345

[B17] Colby T. (2006). SUMO-conjugating and SUMO-deconjugating enzymes from arabidopsis. *Plant Physiol.* 142 318–332. 10.1104/pp.106.085415 16920872 PMC1557612

[B18] Conti L. Nelis S. Zhang C. Woodcock A. Swarup R. Galbiati M. (2014). Small ubiquitin-like modifier protein SUMO enables plants to control growth independently of the phytohormone gibberellin. *Develop. Cell* 28 102–110. 10.1016/j.devcel.2013.12.004 24434138

[B19] Conti L. Price G. O’Donnell E. Schwessinger B. Dominy P. Sadanandom A. (2008). Small ubiquitin-like modifier proteases OVERLY TOLERANT TO SALT1 and -2 regulate salt stress responses in *Arabidopsis*. *Plant Cell* 20 2894–2908. 18849491 10.1105/tpc.108.058669 PMC2590731

[B20] Cruz de Carvalho M. (2008). Drought stress and reactive oxygen species. *Plant Signal. Behav.* 3 156–165. 10.4161/psb.3.3.5536 19513210 PMC2634109

[B21] Cummins I. Cole D. Edwards R. (1999). A role for glutathione transferases functioning as glutathione peroxidases in resistance to multiple herbicides in black-grass. *Plant J.* 18 285–292. 10.1046/j.1365-313X.1999.00452.x 10377994

[B22] Daryanto S. Wang L. Jacinthe P. A. (2017). Global synthesis of drought effects on cereal, legume, tuber and root crops production: a review. *Agric. Water Manag.* 179 18–33. 10.1016/j.agwat.2016.04.022

[B23] de Carvalho K. de Campos M. K. F. Domingues D. S. Pereira L. F. P. Vieira L. G. E. (2013). The accumulation of endogenous proline induces changes in gene expression of several antioxidant enzymes in leaves of transgenic *Swingle citrumelo*. *Mol. Biol. Rep.* 40 3269–3279. 10.1007/s11033-012-2402-5 23292076

[B24] Dehesh K. Klaas M. Huser I. Apel K. (1986). Light-induced changes in the distribution of the 36000-Mr polypeptide of NADPH-protochlorophyllide oxidoreductase within different cellular compartments of barley (*Hordeum vulgare* L.). *Planta* 169 162–171. 10.1007/BF00392310 24232546

[B25] Demirevska K. Zasheva D. Dimitrov R. Simova-Stoilova L. Stamenova M. Feller U. (2009). Drought stress effects on rubisco in wheat: changes in the rubisco large subunit. *Acta Physiol. Plant* 31 1129–1138. 10.1007/s11738-009-0331-2

[B26] Desterro J. Rodriguez M. Kemp G. Hay R. (1999). Identification of the enzyme required for activation of the small ubiquitin-like protein SUMO-1. *J. Biol. Chem.* 274 10618–10624. 10.1074/jbc.274.15.10618 10187858

[B27] Edwards R. Dixon D. Walbot V. (2000). Plant glutathione S-transferases: enzymes with multiple functions in sickness and in health. *Trends Plant Sci.* 5 193–198. 10.1016/S1360-1385(00)01601-0 10785664

[B28] Elrouby N. Coupland G. (2010). Proteome-wide screens for small ubiquitin-like modifier (SUMO) substrates identify *Arabidopsis* proteins implicated in diverse biological processes. *Proc. Acad. Natl. Sci. U.S.A.* 107 17415–17420. 10.1073/pnas.1005452107 20855607 PMC2951436

[B29] Feller U. Anders I. Demirevska K. (2008). Degradation of rubisco and other chloroplast proteins under abiotic stress. *Gen. Appl. Plant Physiol.* 34 5–18.

[B30] Ford K. Cassin A. Bacic A. (2011). Quantitative proteomic analysis of wheat cultivars with differing drought stress tolerance. *Front. Plant Sci.* 2:44. 10.3389/fpls.2011.00044 22639595 PMC3355674

[B31] Foyer C. Lelandais M. Kunert K. (1994). Photooxidative stress in plants. *Physiol. Plantarum* 92 696–717. 10.1111/j.1399-3054.1994.tb03042.x

[B32] Gareau J. Lima C. (2010). The SUMO pathway: emerging mechanisms that shape specificity, conjugation and recognition. *Nat. Rev. Mol. Cell Biol.* 11 861–871. 10.1038/nrm3011 21102611 PMC3079294

[B33] Gholaminr N. Khayatnezhad M. (2011). The effect of end season drought stress on the chlorophyll content, chlorophyll fluorescence parameters and yield in maize cultivars. *Sci. Res. Essays* 6 5351–5357.

[B34] Guerra D. Crosatti C. Khoshro H. Mastrangelo A. Mica E. Mazzucotelli E. (2015). Post-transcriptional and post-translational regulations of drought and heat response in plants: a spider’s web of mechanisms. *Front. Plant Sci.* 6:57. 10.3389/fpls.2015.00057 25717333 PMC4324062

[B35] Hailemichael G. Catalina A. González M. Martin P. (2016). Relationships between water status, leaf chlorophyll content and photosynthetic performance in tempranillo vineyards. *S. Afr. J. Enol.Viti.* 37 149–156. 10.21548/37-2-1004

[B36] Hammad S. Ali O. (2014). Physiological and biochemical studies on drought tolerance of wheat plants by application of amino acids and yeast extract. *Ann. Agric. Sci.* 59 133–145. 10.1016/j.aoas.2014.06.018

[B37] Hansen L. Van den Burg H. Van Ooijen G. (2017). SUMOylation contributes to timekeeping and temperature compensation of the plant circadian clock. *J. Biol. Rhytm.* 32 560–569. 10.1177/0748730417737633 29172926

[B38] Hoque M. A. O. E. Banu M. N. A. Nakamura Y. Shimoishi Y. Murata Y. (2007). Exogenous proline mitigates the detrimental effects of salt stress more than the betaine by increasing antioxidant enzyme activities. *J. Plant Physiol.* 164 553–561. 10.1016/j.jplph.2006.03.010 16650912

[B39] Hossain M. A. Hasanuzzaman M. Fujita M. (2011). Coordinate induction of anti-oxidant defense and glyoxalase system by exogenous proline and glycinebetaine is correlated with salt tolerance in mung bean. *Front. Agric. China* 5 1–14. 10.1007/s11703-010-1070-2

[B40] Ihsan M. Z. El-Nakhlawy F. S. Ismail S. M. Fahad S. (2016). Wheat phenological development and growth studies as affected by drought and late season high temperature stress under arid environment. *Front. Plant Sci.* 7:795. 10.3389/fpls.2016.00795 27375650 PMC4893551

[B41] Johnson E. (2004). Protein Modification by SUMO. *Annu. Rev. Biochem.* 73 355–382. 10.1146/annurev.biochem.73.011303.074118 15189146

[B42] Khanna-Chopra R. (2011). Leaf senescence and abiotic stresses share reactive oxygen species-mediated chloroplast degradation. *Protoplasma* 249 469–481. 10.1007/s00709-011-0308-z 21805384

[B43] Kidrič M. Kos J. Sabotič J. (2014). Proteases and their endogenous inhibitors in the plant response to abiotic stress. *Bot. Serbica* 38 139–158.

[B44] Lawlor D. W. (2012). Genetic engineering to improve plant performance under drought: physiological evaluation of achievements, limitations, and possibilities. *J. Exp. Bot.* 64 83–108. 10.1093/jxb/ers326 23162116

[B45] Le Roux M. L. (2015). *Engineering wheat (Triticum aestivum L.) for abiotic resilience by manipulating small ubiquitin-like modifiers.* M.Sc. thesis, Stellenbosch University, South Africa.

[B46] Liu L. Yan X. Kong X. Zhao Y. Gong Z. Jin J. (2016). Transcriptional gene silencing maintained by OTS1 SUMO protease requires a dna-dependent polymerase V-Dependent Pathway. *Plant Physiol.* 173 655–667. 10.1104/pp.16.01365 27852949 PMC5210737

[B47] Mafakheri A. B. Siosemardeh P. C. Bahramnejad Y. Struik T. Sohrabi S. (2010). Effect of drought stress on yield, proline and chlorophyll contents in three chickpea cultivars. *Aust. J. Crop Sci.* 4 580–585.

[B48] Marcus Y. Altman-Gueta H. Snir A. Wolff Y. Gurevitz M. (2008). “Does Rubisco limit the rate of photosynthesis? Chapter 3,” in *Photosynthesis. Energy from the Sun: 14th International Congress on Photosynthesis*, eds Allen J. F. Gantt E. Golbeck J. H. Osmond B. (Berlin: Springer), 863–866.

[B49] Marrs K. (1996). The functions and regulation of glutathione S-transferases in plants. *Ann. Revi. Plant Physiol. Plant Mol. Biol.* 47 127–158. 10.1146/annurev.arplant.47.1.127 15012285

[B50] Matsumoto K. Mizoue K. Kitamura K. Tse W. C. Huber C. P. Ishida T. (1999). Structural basis of inhibition of cysteine proteases by E-64 and its derivatives. *Biopolymers* 51 99–107. 10.1002/(SICI)1097-0282(1999)51:1<99::AID-BIP11>3.0.CO;2-R 10380357

[B51] Miura K. Hasegawa P. M. (2009). Sumoylation and abscisic acid signaling. *Plant Signal. Behav.* 4 1176–1178. 20514240 10.4161/psb.4.12.10044 PMC2819450

[B52] Montagu K. Woo K. (1999). Recovery of tree photosynthetic capacity from seasonal drought in the wet–dry tropics: the role of phyllode and canopy processes in *Acacia auriculiformis*. *Aust. J. Plant Physiol.* 26 135–145. 10.1071/PP98034

[B53] Motulsky H. J. (2014). Common misconceptions about data analysis and statistics. *Naunyn Schmiedebergs Arch. Pharmacol.* 387 1017–1023. 10.1007/s00210-014-1037-6 25213136 PMC4203998

[B54] Nilsen E. T. Orcutt D. M. (1996). *Physiology of Plants Under Stress: Abiotic Factors*, 2nd Edn. New York, NY: John Wiley and Sons Inc.

[B55] Paknejad F. Nasri M. Moghadam H. Zahedi H. Alahmadi M. (2007). Effects of drought stress on chlorophyll fluorescence parameters, chlorophyll content and grain yield of wheat cultivars. *J. Biol. Sci.* 7 841–847. 10.3923/jbs.2007.841.847

[B56] Palma J. Sandalio L. Javier Corpas F. Romero-Puertas M. McCarthy I. del Río L. (2002). Plant proteases, protein degradation, and oxidative stress: role of peroxisomes. *Plant Physiol. Biochem.* 40 521–530. 10.1016/S0981-9428(02)01404-3

[B57] Parkhill J. Maillet G. Cullen J. (2001). Fluorescence-based maximal quantum yield for PSII as a diagnostic of nutrient stress. *J. Phycol.* 37 517–529. 10.1046/j.1529-8817.2001.037004517.x

[B58] Perdomo J. Capó-Bauçà S. Carmo-Silva E. Galmés J. (2017). Rubisco and rubisco activase play an important role in the biochemical limitations of photosynthesis in rice, wheat, and maize under high temperature and water deficit. *Front. Plant Sci.* 8:490. 10.3389/fpls.2017.00490 28450871 PMC5390490

[B59] Rampino P. Pataleo S. Gerardi C. Mita G. Perrotta C. (2006). Drought stress response in wheat: physiological and molecular analysis of resistant and sensitive genotypes. *Plant Cell Environ.* 29 2143–2152. 10.1111/j.1365-3040.2006.01588.x 17081248

[B60] Raunser S. Magnani R. Huang Z. Houtz R. Trievel R. Penczek P. (2009). Rubisco in complex with rubisco large subunit methyltransferase. *Proc. Natl. Acad. Sci. U.S.A.* 106 3160–3165. 10.1073/pnas.0810563106 19208805 PMC2638739

[B61] Reeves P. Murtas G. Dash S. Coupland G. (2002). Early in short days 4, a mutation in Arabidopsis that causes early flowering and reduces the mRNA abundance of the floral repressor FLC. *Development* 129 5349–5361. 10.1242/dev.00113 12403707

[B62] Rejeb K. B. Abdelly C. Savouré A. (2014). How reactive oxygen species and proline face stress together. *Plant Physiol. Biochem.* 80 278–284. 10.1016/j.plaphy.2014.04.007 24813727

[B63] Roxas V. Lodhi S. Garrett D. Mahan J. Allen R. (2000). Stress tolerance in transgenic tobacco seedlings that overexpress glutathione S-transferase/Glutathione peroxidase. *Plant Cell Physiol.* 41 1229–1234. 10.1093/pcp/pcd051 11092907

[B64] Rylatt D. Parish C. (1982). Protein determination on an automatic spectrophotometer. *Anal. Biochem.* 121 213–214. 10.1016/0003-2697(82)90578-4 7091680

[B65] Rytz T. C. Miller M. J. McLoughlin F. Augustine R. C. Marshall R. S. Juan Y.-T. (2018). SUMOylome profiling reveals a diverse array of nuclear targets modified by the SUMO ligase SIZ1 during heat stress. *Plant Cell* 30 1077–1099. 10.1105/tpc.17.00993 29588388 PMC6002191

[B66] Sade D. Sade N. Shriki O. Lerner S. Gebremedhin A. Karavani A. (2014). Water balance, hormone homeostasis, and sugar signaling are all involved in tomato resistance to tomato yellow leaf curl virus. *Plant Physiol.* 165 1684–1697. 10.1104/pp.114.243402 24989233 PMC4119048

[B67] Sade N. Galkin E. Moshelion M. (2015). Measuring arabidopsis, tomato and barley leaf relative water content (RWC). *BioProtoco* 5:e1451 10.21769/BioProtoc.1451.l

[B68] Saracco S. Miller M. Kurepa J. Vierstra R. (2007). Genetic analysis of SUMOylation in Arabidopsis: conjugation of SUMO1 and SUMO2 to nuclear proteins is essential. *Plant Physiol.* 145 119–134. 10.1104/pp.107.102285 17644626 PMC1976578

[B69] Seki M. Narusaka M. Ishida J. Nanjo T. Fujita M. Oono Y. (2002). Monitoring the expression profiles of 7000 Arabidopsis genes under drought, cold and high-salinity stresses using a full-length cDNA microarray. *Plant J.* 31 279–292. 10.1046/j.1365-313X.2002.01359.x 12164808

[B70] Shavrukov Y. Kurishbayev A. Jatayev S. Shvidchenko V. Zotova L. Koekemoer F. (2017). Early flowering as a drought escape mechanism in plants: how can it aid wheat production? *Front. Plant Sci.* 8:1950. 10.3389/fpls.2017.01950 29204147 PMC5698779

[B71] Srivastava A. Zhang C. Caine R. Gray J. Sadanandom A. (2017). Rice SUMO protease overly tolerant to Salt 1 targets the transcription factor, OsbZIP23 to promote drought tolerance in rice. *Plant J.* 92 1031–1043. 10.1111/tpj.13739 29024118

[B72] Srivastava A. Zhang C. Sadanandom A. (2016a). Rice overly tolerant to salt 1 (OTS1) SUMO protease is a positive regulator of seed germination and root development. *Plant Signal. Behav.* 1:e1173301. 10.1080/15592324.2016.1173301 27119209 PMC4973764

[B73] Srivastava A. Zhang C. Yates G. Bailey M. Brown A. Sadanandom A. (2016b). SUMO Is a critical regulator of salt stress responses in rice? *Plant Physiol.* 170 2378–2391. 10.1104/pp.15.01530 26869703 PMC4825142

[B74] Strasser R. J. Tsimilli-Michael M. Srivastava A. (2004). “Analysis of the fluorescence transient,” in *Chlorophylla Fluorescence: a Signature of Photosynthesis*, eds Papageorgiou G. C. Govindjee (Dordrecht: Springer), 321–362.

[B75] Vendruscolo E. C. G. Schuster I. Pilegg M. Scapim C. A. Molinari H. B. C. Marur C. J. (2007). Stress-induced synthesis of proline confers tolerance to water deficit in transgenic wheat. *J. Plant Physiol.* 164 1367–1376. 10.1016/j.jplph.2007.05.001 17604875

[B76] Venisse J. S. Gullner G. Brisset M. N. (2001). Evidence for the involvement of an oxidative stress in the initiation of infection of pear by *Erwinia amylovora*. *Plant Physiol.* 125 2164–2172. 10.1104/pp.125.4.2164 11299395 PMC88871

[B77] Verma V. Croley F. Sadanandom A. (2018). Fifty shades of SUMO: its role in immunity and at the fulcrum of the growth-defence balance. *Mol. Plant Pathol.* 19 1537–1544. 10.1111/mpp.12625 29024335 PMC6637990

[B78] Witt S. Galicia L. Lisec J. Cairns J. Tiessen A. Araus J. (2012). Metabolic and phenotypic responses of greenhouse-grown maize hybrids to experimentally controlled drought stress. *Mol. Plant* 5 401–417. 10.1093/mp/ssr102 22180467

[B79] Zadoks J. C. Chang T. T. Konzak C. F. (1974). A decimal code for the growth stages of cereals. *Weed Res.* 14 415–421. 10.1111/j.1365-3180.1974.tb01084.x

[B80] Zampieri M. Ceglar A. Dentener F. Toreti A. (2017). Wheat yield loss attributable to heat waves, drought and water excess at the global, national and subnational scales. *Environ. Res. Lett.* 12:e064008 10.1088/1748-9326/aa723b

[B81] Zarco-Tejada P. J. Miller J. R. Mohammed G. H. Noland T. L. Sampson P. H. (2000). Chlorophyll fluorescence effects on vegetation apparent reflectance: II. Laboratory and Airborne canopy-level measurements with hyperspectral data. *Remote Sens. Environ.* 74 596–608. 10.1016/S0034-4257(00)00149-8

[B82] Zhang J. Zhang S. Cheng M. Jiang H. Zhang X. Peng C. (2018). Effect of drought on agronomic traits of rice and wheat: a meta-analysis. *Int. J. Environ. Res. Public Health* 15:E839. 10.3390/ijerph15050839 29695095 PMC5981878

[B83] Zieslin N. Ben-Zaken R. (1991). Peroxidase, phenylalanine ammonia-lyase and lignification in peduncles of rose flowers. *Plant Physiol. Biochem.* 29 147–151.

