# Supplementary material for: Expression of a Small Ubiquitin-Like Modifier Protease Increases Drought Tolerance in Wheat (Triticum aestivum L.)
Source: Front Plant Sci. 2019 Mar 8;10:266. doi: 10.3389/fpls.2019.00266 (PMC6418343; doi:10.3389/fpls.2019.00266)
Supplement: Supplementary file 1 [file Table_1.DOCX]

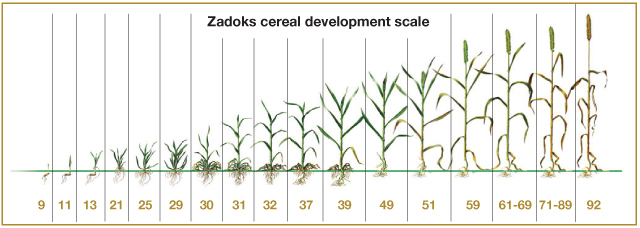


**Figure S1** Wheat developmental stages until maturity (Zadoks cereal development scale; Zadoks *et al*., 1974; Vendruscolo *et al*., 2007).
