# Supplementary figures and images for: Expression of a Small Ubiquitin-Like Modifier Protease Increases Drought Tolerance in Wheat (Triticum aestivum L.)
Source: Front Plant Sci. 2019 Mar 8;10:266. doi: 10.3389/fpls.2019.00266 (PMC6418343; doi:10.3389/fpls.2019.00266)

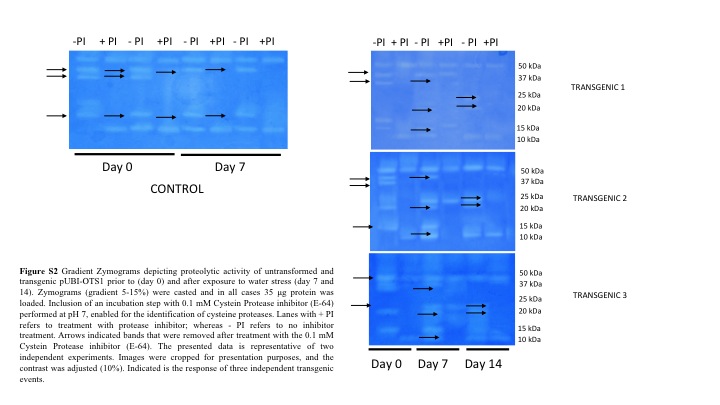

Supplement: Supplementary file 2 [file Image_1.JPEG]
